# Experimental design of a culture approach for corneal endothelial cells of New Zealand white rabbit

**DOI:** 10.1016/j.heliyon.2020.e05178

**Published:** 2020-10-06

**Authors:** María Dolores Montalvo-Parra, Isaac Alejandro Vidal-Paredes, Cesar E. Calzada-Rodríguez, Italia Tatnaí Cárdenas-Rodríguez, Guiomar Farid Torres-Guerrero, Daniela Gómez-Elizondo, Mariana López-Martínez, Judith Zavala, Jorge E. Valdez-García

**Affiliations:** Tecnologico de Monterrey, Escuela de Medicina y Ciencias de la Salud, 3000 Morones Prieto Ave., Col. Los Doctores, Monterrey, N.L., Mexico

**Keywords:** Tissue engineering, Cell culture, Cell differentiation, Extracellular matrix, Tissue culture, Cell biology, Regenerative medicine, Ophthalmology, Corneal endothelium, ATPase, Collagen, Circularity, Experimental design

## Abstract

The harvesting of corneal endothelial cells (CEC) has received special attention due to its potential as a therapy for corneal blindness. The main challenges are related to the culture media formulation, cellular density at the primary isolation, and the number of passages in which CEC can retain their functional characteristics. To alternate different media formulations to harvest CEC has an impact on the cellular yield and morphology. Therefore, we analyzed four different sequences of growth factor-supplemented Stimulatory (S) and non-supplemented Quiescent (Q) media, upon passages to find the optimal S-Q culture sequence. We assessed cell yield, morphology, procollagen I production, Na^+^/K^+^-ATPase function, and the expression of ZO-1 and Na^+^/K^+^-ATPase. Our results show SQSQ and SQQQ sequences with a balance between an improved cell yield and hexagonal morphology rate. CEC cultured in the SQQQ sequence produced procollagen I, showed Na^+^/K^+^-ATPase function, and expression of ZO-1 and Na^+^/K^+^-ATPase. Our study sets a culture approach to guarantee CEC expansion, as well as functionality for their potential use in tissue engineering and in vivo analyses. Thus, the alternation of S and Q media improves CEC culture. SQQQ sequence demonstrated CEC proliferation and lower the cost implied in SQSQ sequences. We discarded the use of pituitary extract and ROCK inhibitors as essential for CEC proliferation.

## 1 Introduction

Corneal endothelial cells (CEC) are the cells of the inner layer of the cornea that functions as a barrier and pump for the maintenance of the optimal hydration level for the eye to perform its vision function [1, 2]. The clarity and nutrition of the cornea rely on these functions, given that it is an avascular tissue [3]. Hence, CEC loss produced can lead to blindness due to a series of concatenated events; starting by destabilization of the endothelial barrier, subsequent inability to pump fluid out of the stroma, stromal edema, and loss of transparency [4, 5]. Moreover, CEC is arrested in the G_1_ cell cycle phase [6] thus, repair after cell density loss is limited [7, 8]. Corneal blindness can only be treated through transplantation, however, this procedure faces the shortage of tissue donors [3, 9, 10, 11, 12].

The expansion of CEC through culture has received special attention among the efforts to develop an alternative to donor tissue. Several experimental approaches aim to develop a method to isolate and harvest CEC for corneal blindness cell therapy and tissue engineering [3, 13].

For these purposes, challenges such as culture media [1, 14, 15, 16, 17] formulation cellular density [18], and the number of passages that assures functionality [17, 19] need to be overcome. The factors that affect the overall yield of CEC are donor-to-donor variation and the use of different supplements in culture media [20, 21, 22]. Among the supplements used are pituitary extract, an exogenous and complex element, and Rho-associated kinase (ROCK) inhibitors that mediate several cellular functions. In CEC culture, media with pituitary extract is reported to promote proliferative capacity [15], and so do ROCK inhibitors. Moreover, although a robust and clearly described culture methodology is still lacking [23], a great amount of clinical interest has been generated for the development of alternative approaches in the treatment of corneal endothelium damage-associated corneal blindness using cultured CEC.

We previously reported a two-phase CEC culture system that allows cellular expansion and retains specific molecular markers, hexagonal morphology, and monolayer arrangement [24]. Now, to set the best culture media combination, we aimed to analyze the CEC obtained from different approaches in terms of cell yield, morphological analysis, pro-collagen production, Na^+^/K^+^-ATPase function, and the expression of ZO-1 and Na^+^/K^+^-ATPase.

## 2 Methods

### 2.1 Isolation and experimental design of CEC culture

This study was approved by the Institutional Ethics Committee (School of Medicine of Tecnologico de Monterrey), number 2017-005. All animals were treated according to the Guide for the Care and Use of Laboratory Animals.

The corneas were obtained from four 3-month-old New Zealand rabbits weighing ~3 kg [25, 26]. The rabbits were euthanized under general anesthesia with a combination of xylazine 5 mg/kg and 30 mg/kg of ketamine (Pisa Farmacéutica, C866M60, Guadalajara, JAL, México); followed by a lethal intraperitoneal injection of sodic pentobarbital (Pets Pharma, Q7972004 Nezahualcoyotl, MEX, Mexico). The corneas were excised, rinsed with 37 °C Phosphate Buffered Saline (PBS) pH 7.4 (Gibco, Thermo Fisher Scientific, 10010023, Waltham, MA, USA) with 1% penicillin/streptomycin (Pen/Strep; Thermo Fisher Scientific, 15140148, Grand Island, NY, USA), and placed in a sterile tissue culture dish.

All rabbit CEC were isolated using the “peel-and-digest” approach. Excised whole corneas were placed in a petri dish with PBS, all inside the Biosafety cabinet. Then, Trypan blue 0.04% (Millipore Sigma, T8154, Darmstadt, HE, Germany) was poured on the endothelial side of the cornea for 1 min to stain corneal endothelium borders. Trypan blue was then rinsed using PBS. The border of CE was gently pushed around the whole circumference. Descemet's membrane with the intact endothelium was carefully peeled from the corneal stroma and rinsed several times with 37 °C PBS with 1% antibiotics, then incubated in Opti-MEM I 8% FBS and 1% antibiotic overnight to stabilize the cells before culture. CEC were incubated, while shaking at 170, rpm with 1 mg/ml of collagenase (Sigma-Aldrich Co., C0130, St. Louis, MO, USA) at 37 °C for 1 h. Cells were collected following centrifugation at 375 × g for 10 min and seeded into a 12-well culture dish.

**Figure 1:**
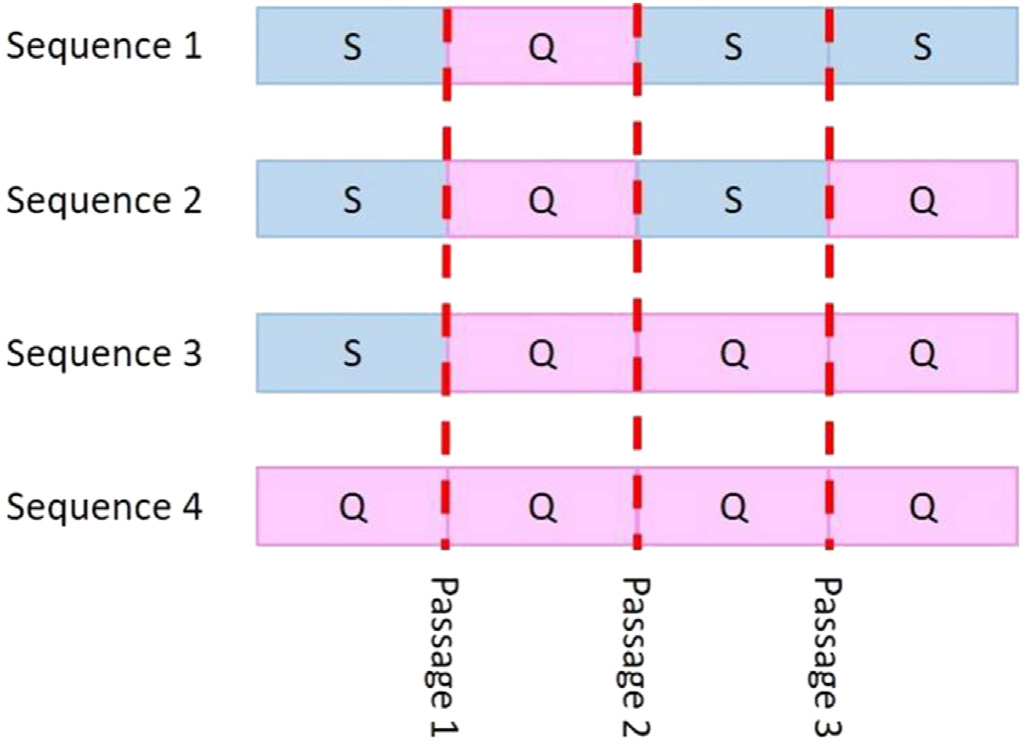
Experimental design. Four sequences of alternating Stimulating (S) and Quiescent (Q) were evaluated. All cultures were stabilized with proliferative media until confluence at passage 0; QQQQ sequence was the exception. Passages are marked with dashed red lines.

A two-phase culture system was established to be used in CEC culture. Two-phase refers to the alternation of two media of different compositions: Stimulatory (S) and quiescent (Q) media. Our modified formula of previously reported Stimulatory media [15, 27], eliminates the use of pituitary extract and contained Opti-MEM I (Gibco®; Thermo Fisher Scientific, Waltham, MA, USA) supplemented with 8% fetal bovine serum (FBS; Cellgro, C866M60, Manassas, VA, USA) and 1% antibiotic (Pen/Strep; Thermo Fisher Scientific, 15140148, Grand Island, NY, USA), 20 ng/ml of nerve growth factor (NGF; Sigma-Aldrich Co., 9061614, Saint Louis, MO, USA), 5 ng/ml of epidermal growth factor (EGF; Sigma-Aldrich Co., GF316, Saint Louis, MO, USA), 200 mg/l of calcium chloride (Sigma-Aldrich Co., 10043524, Saint Louis, MO, USA), 20 μg/ml of ascorbic acid (Sigma-Aldrich Co., A92902, Saint Louis, MO, USA), 0.08% chondroitin sulfate (Sigma-Aldrich Co., C4384, Saint Louis, MO, USA). Quiescent media formulation was Opti-MEM I supplemented with 8% FBS and 1% antibiotic. The experimental design consisted of alternating media through several passages to evaluate combinations of the phases of our culture system as shown in Figure 1, four sequences of alternating media were evaluated. The objective was to produce a maximum cell yield while conserving CEC characteristics.

Passage 0 consisted of recently isolated CEC cultured in a 12-well plate with Stimulatory media until hexagonal morphology was observed (confluence beyond 80%). All passages herein mentioned followed a hexagonal morphology and >80% confluence rule. At passage 1, CEC were split 1:2 and the medium was changed using Q media. The culture continued up to passage 3. An Axiovert 40 CFL contrast microscope (CFL; Carl Zeiss AG, Oberkochen, ZWS, Germany) featuring a PowerShot A640 digital camera (Canon Inc., Tokyo, Japan) was used to register the cell morphology.

### 2.2 Cell yield analysis

Cell yield was calculated to produce evidence to select the media sequence with a higher yield. Using the raw cell count data at Table 1, expansion folds were calculated (Table 2) as the quotient of final cellular concentration over initial cellular concentration for each passage and media sequence. We also calculated a final fold between passages 3 and 1.

**Table 1:** Media sequence selection based on cellular expansion yield. Higher cell yields were reached with sequences 1–7. Cellular size was observed to be smaller at higher yields. Selection of optimum sequence also considered morphology. Thus, sequence 4 (SQQQ) was selected. Expansion yield was calculated for each media sequence as the average of the difference between the initial seeded cells and the final cellular concentration upon passage. Cell counts were performed with a Neubauer chamber by duplicate. Stimulatory media (S), Quiescent media (Q).

Cell concentration (cell number × 10^3^)/mm^3^						
Sequence	Passage 1		Passage 2		Passage 3	
	Initial	Final	Initial	Final	Initial	Final
1) P-R-P-P-R	15	60		126		190
2) P-R-P-P-R	23	73		142		150
3) P-R-P-R	30	80		133		140
4) P-R-R-R	17	63		80		128
5) P-R-R-R	13	58		83		50
6) P-R-R-R	19	62		82		97
7) P-R-R-R	22	26		80		28
8) R-R-R-R	26	40		52		68

**Table 2:** Passage fold change analysis. A fold change ratio from passage to passage was calculated to ease the comparison of cell yield within passages. Final fold = passage 3/passage 1.

Fold change (Δ)				
Sequence	Passage 1	Passage 2	Passage 3	Final Fold
1) S-Q-S-S-Q	4	2.1	1.58	12.6
2) S-Q-S-S-Q	3.2	1.9	1.05	6.5
3) S-Q-S-Q	2.6	1.9	1.05	4.6
4) S-Q-Q-Q	3.7	1.3	1.6	7.5
5) S-Q-Q-Q	4.46	1.43	0.6	3.84
6) S-Q-Q-Q	3.26	1.32	1.8	5.1
7) S-Q-Q-Q	0.18	1.15	0.93	1.27
8) Q-Q-Q-Q	1.53	1.3	1.3	2.61

### 2.3 Morphological analysis

CEC were observed (Axiovert 40CFL inverted microscope, Zeiss, Oberkochen, ZWS, Germany) and photo-documented daily. The scale was set on each photograph and 40 cells per daily image were delimited with free shape ROI tool from Image J. Perimeter, area, circularity, aspect ratio, and roundness indexes were obtained using Measure (Analyze menu). Basal parameters were also measured from cells observed in pictures obtained from recently isolated Descemet's membrane. Microsoft Excel (2007, Redmond, WA, USA) was used for data processing. Statistical analysis and graph creation were carried out using Systat Sigma Plot (V. 11, San Jose, CA, USA).

### 2.4 Protein production analysis

CEC at passage 2, cultured with SQQQ sequence, were detached using trypsin and then centrifuged at 500 x g for 5 min at 4 °C to obtain a cell lysate for total protein analysis. The pellet was washed three times with 1X PBS buffer and incubated 20 min with an extraction buffer (Abcam, ab193970, Cambridge, MA, USA) on ice. The solution was centrifuged at 18,000 x g for 20 min at 4 °C. The supernatant was used for the total protein concentration analysis. Colorimetric bicinchoninic acid assay (BCA; Pierce 23225, Rockford, IL, USA) was used. A standard curve was prepared using albumin provided in the kit. The concentration of total protein was measured at 562 nm in a spectrophotometer. The assay was done in triplicates using Human Dermal Fibroblasts adult (HDFa) line as control. Statistical analysis was performed with a *t*-test using Microsoft Excel (2007, Redmond, WA, USA).

### 2.5 Procollagen I-α1 analysis

Procollagen I-α1 was determined as a measure of the ability of the cells to produce collagen 1-α1, ELISA Kit (Abcam, ab210966, Cambridge, MA, USA) was used with the cell lysates described before. The procedure was followed following the kit manufacturer and the absorbance was measured at 562 nm in a microplate reader. The assay was done in triplicates on CEC passage 2 cultured with SQQQ sequence using HDFa as control. Statistical analysis was performed with a *t*-test using Microsoft Excel (2007, Redmond, WA, USA).

### 2.6 Biomarker immunodetection (ZO1 and Na^+^/K^+^-ATPase)

Immunocytochemistry was performed on the basal whole cornea and on CEC passage 2 cultured with SQQQ sequence to analyze the presence ZO-1 (ThermoFisher, 617300, Waltham, MA, USA), and Na^+^/K^+^-ATPase (Abcam, ab176163, Cambridge, MA, USA). The procedure consisted of overnight cell stabilization over coverslips with poly-D lysine (Sigma-Aldrich, P7280, Saint Louis, MO, USA), fixation with 4% paraformaldehyde, nonspecific bonding blockage with 5% bovine serum albumin (BSA; Sigma-Aldrich, A7030, Saint Louis, MO, USA), overnight 4 °C incubation with primary antibodies (ZO-1 5 μg/ml and Na^+^/K^+^-ATPase 1:100), and incubation with Alexa Fluor 488 secondary antibody (Abcam, ab150077, Cambridge, MA, USA; 2 μg/ml) for 1 h at room temperature. Fluroshield Mounting Medium with 4′, 6-Diamidino-2′-phenylindole dihydrochloride (DAPI; Abcam, ab104139, Cambridge, MA, USA) counterstain was used 10 μl per coverslip (0.1–1 μg/mL). Epifluorescence was registered with a wide-field fluorescence microscope (Zeiss Imager Z1) with an AxioCam HRm (Zeiss) camera (Göttingen, NI, Germany). Whole width cornea sections were used as controls [6].

### 2.7 Na^+^/K^+^-ATPase activity analysis

A colorimetric enzymatic assay was used to measure the Na^+^/K^+^-ATPase activity (MyBioSource, MBS8243226, San Diego, CA, USA). CEC at passage 2 cultured with SQQQ sequence*,* and HDFa were harvested when confluence was reached and sonicated for the detection per the manufacturer procedure. The final absorbance was registered at 660 nm. The assay was done in triplicates using HDFa as control. Statistical analysis was performed with a *t*-test using Microsoft Excel (2007, Redmond, WA, USA).

## 3 Results

### 3.1 Cell yield

**Figure 2:**
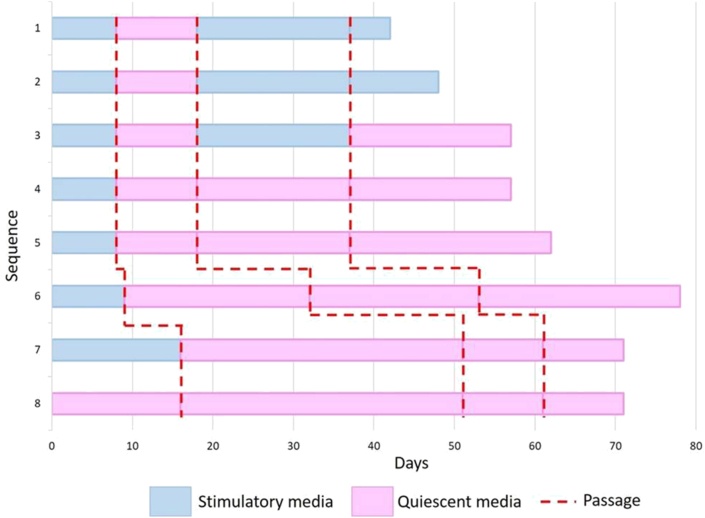
Media alternation sequence design. The first approach to optimum media sequence was determined through this experimental design. Passage number was experimental as in a screening study to corroborate literature. Stimulatory media (S); Quiescent media (Q).

Sequences SQSSQ produced a 12-fold and 6.5-fold, SQSQ sequence a 4.6-fold, and SQQQ sequence produced a 7.5-fold, 3.84-fold, 5.1-fold and 1.27-fold (Table 2). Time to confluence varied for the different media sequences as shown in Figure 2. Moreover, differences in the time to reach confluence were observed in the same sequence as seen for combinations SQQQ.

### 3.2 Cell morphology

**Figure 3:**
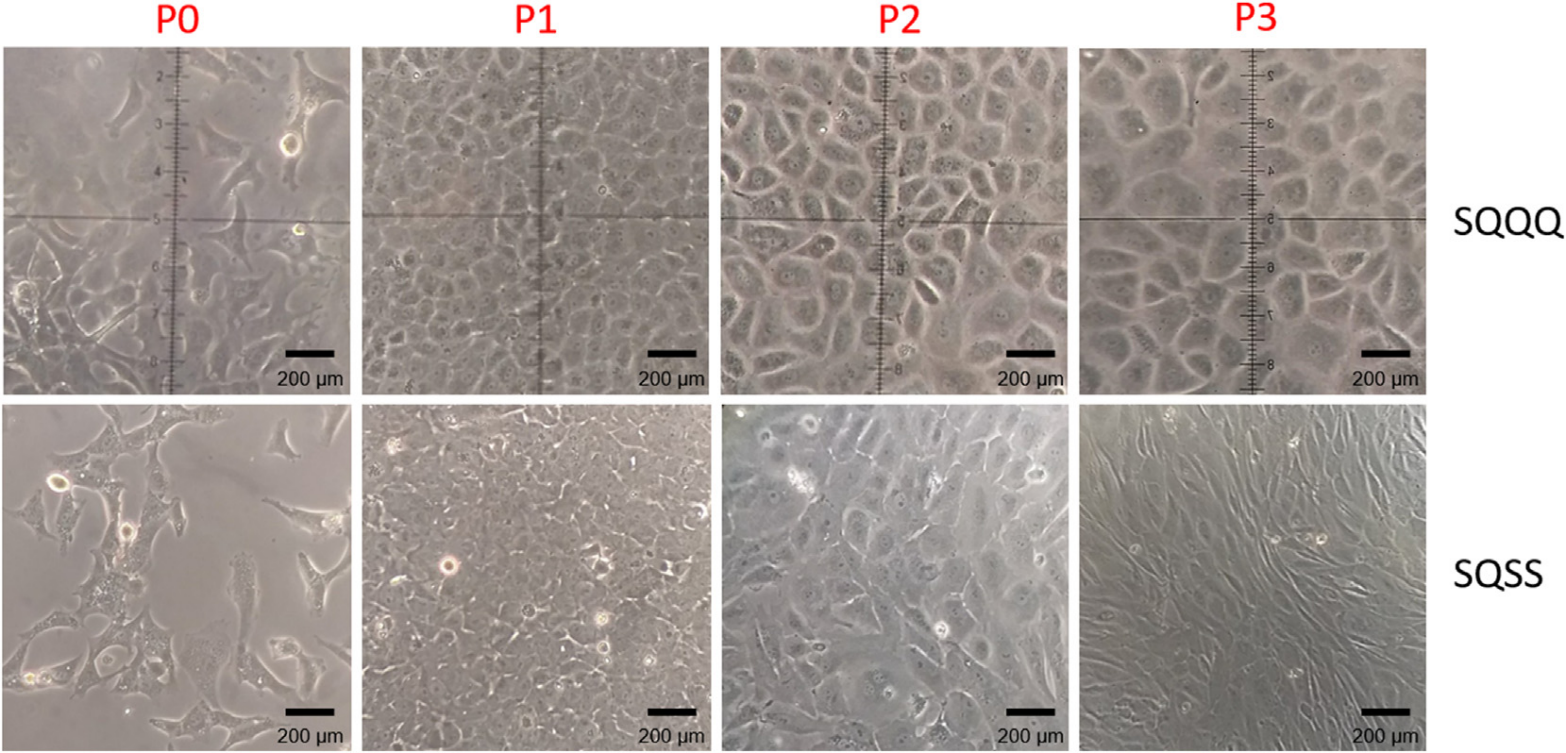
Morphology comparison of CEC cultured with two different sequences. P0 marks the initial state of culture. Passage 1 (P1) through passage 3 (P3) correspond to final point before the following passage. Stimulating (S) and Quiescent (Q) were evaluated.

**Figure 4:**
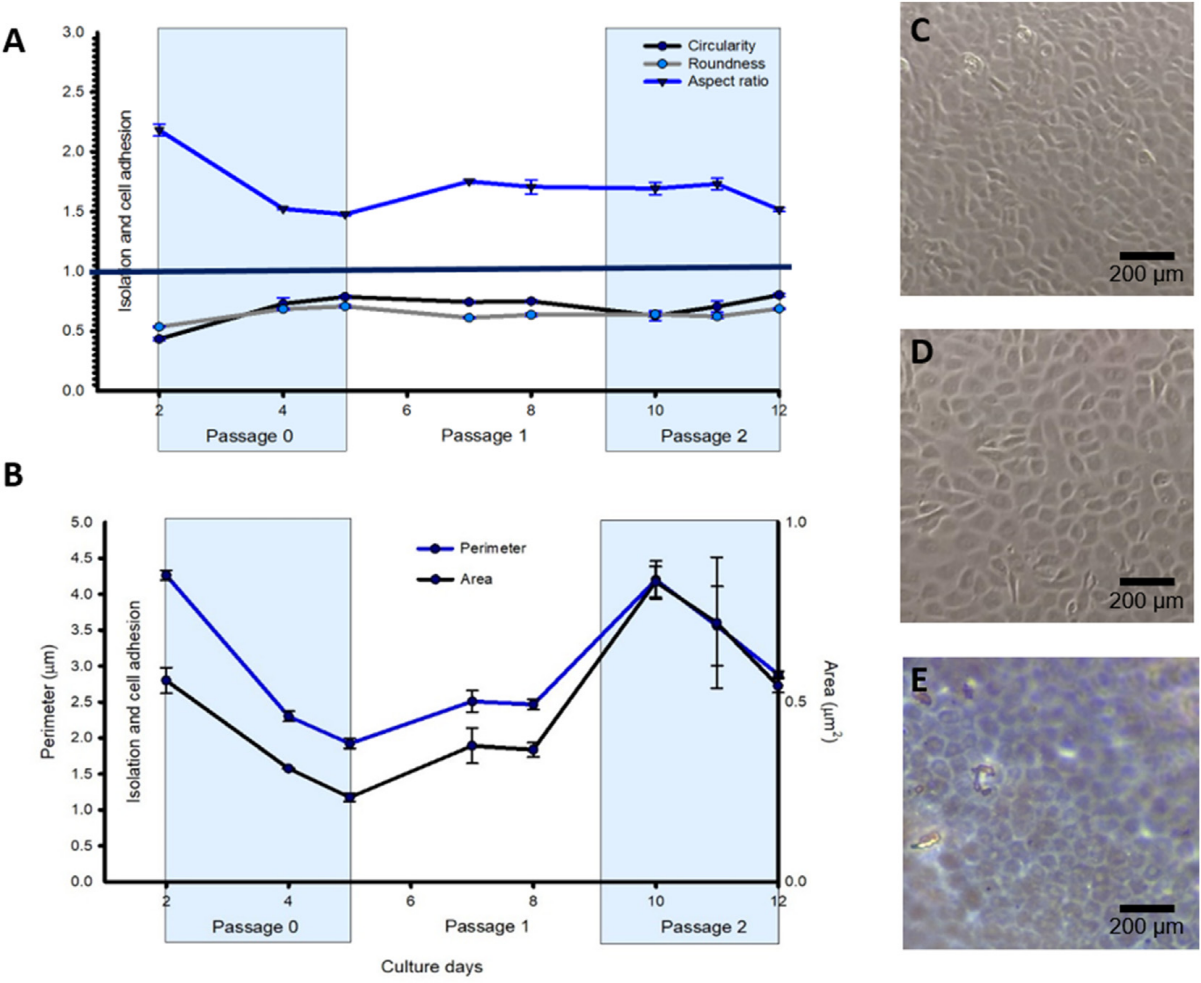
Culture of rabbit corneal endothelium cells with SQQQ combination. A) Changes through time of shape descriptors: circularity (p < 0.001), roundness (p < 0.001) and aspect ratio (p > 0.001); B) changes in cell area (p > .001) and perimeter (p < .001); C) rabbit corneal endothelium cells at passage 0, day 3; D) rabbit corneal endothelium cells at passage 2; E) recently isolated Descemet's membrane. (N = 3; p = 0.001).

We previously reported circularity values for all-proliferative-media combination (0.41 ± 0.19 SD), and circularity control values of primary isolated rabbit CEC (0.77 ± 0.063 SD) [24]. Preliminary results comparing the morphology changes between two sequences (SQQQ and SQSS) throughout three passages are shown in Figure 3. Our present result on circularity evaluation of the QQQQ sequence was 0.69 ± 0.11 SD. Sequences with SQSQ combination showed a 0.60 ± 0.14 SD circularity value, and SQQQ media combination 0.78 ± 0.045 SD at the end of passage 2 (Figure 4A). Sequences SQSSQ were not evaluated for these parameters due to the extreme loss of uniformity on the monolayer. So, SQQQ was selected as the balance between circularity and a good proliferation rate. Then, we further evaluated roundness, aspect ratio, area, and perimeter in the selected sequence through three passages to assess morphological changes as shown in Figure 4. The value of 1 represents a perfect circle and the obtained results for Circularity (median 0.71; final value 0.78 ± 0.045 SD), Roundness (median 0.63; final value 6.68 ± 0.043 SD), and Aspect Ratio (median 1.56; final value 1.49 ± 0.127 SD) tend to 1 representing polygonal shapes [27]. In addition, there is a change in area and perimeter upon confluence (Figure 4B). The observed drop in these values indicates that cell size is affected by contact inhibition.

### 3.3 Protein concentration, pro-collagen I α1 production, and Na^+^/K^+^-ATPase analysis

**Figure 5:**
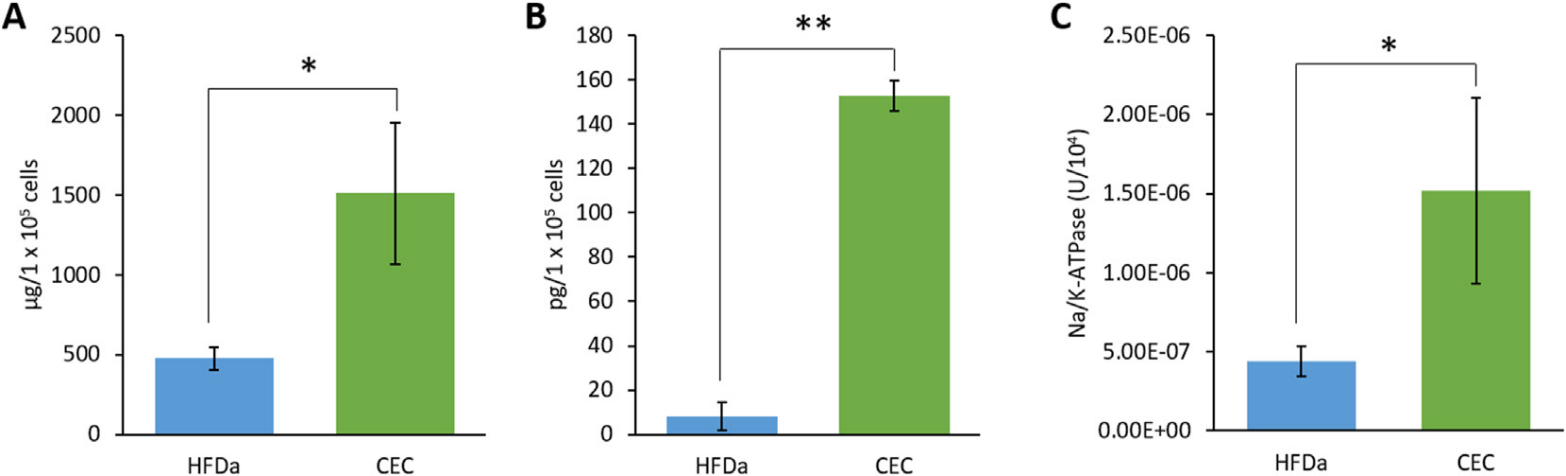
Protein production analysis of rabbit corneal endothelium cells with a SQQQ combination. Total protein concentration A), procollagen I B), and Na+/K + -ATPase c) in Human Dermal Fibroblasts adult (HDFa) and CEC cultured with the SQQQ sequence (∗p < 0.05, ∗∗p < 0.01).

Total protein concentration for CEC was 1,511.02 ± 443.19 μg/1 × 10^5^ and 490 ± 70.26 μg/1 × 10^5^ for HDFa. Figure 5A shows the total protein concentration μg per 1 × 10^5^ cells. A significant difference with a P-value <0.05 was found between the CEC and control cells. Procollagen I determination showed a significant difference between CEC and control cells with a P-value <0.01 (Figure 5B). Procollagen I was also found to represent less than 1% of the total protein of corneal endothelial cell lysates (9.94 × 10-6 %) and was found to be 6 times higher compared to control cells. Na^+^/K^+^-ATPase concentration was 1.52 × 10^−6^ U/10^4^ in CEC and 4.42 × 10^−7^ U/10^4^ in HDFa (Figure 5C).

### 3.4 Biomarker immunodetection (ZO1 and Na^+^/K^+^-ATPase)

**Figure 6:**
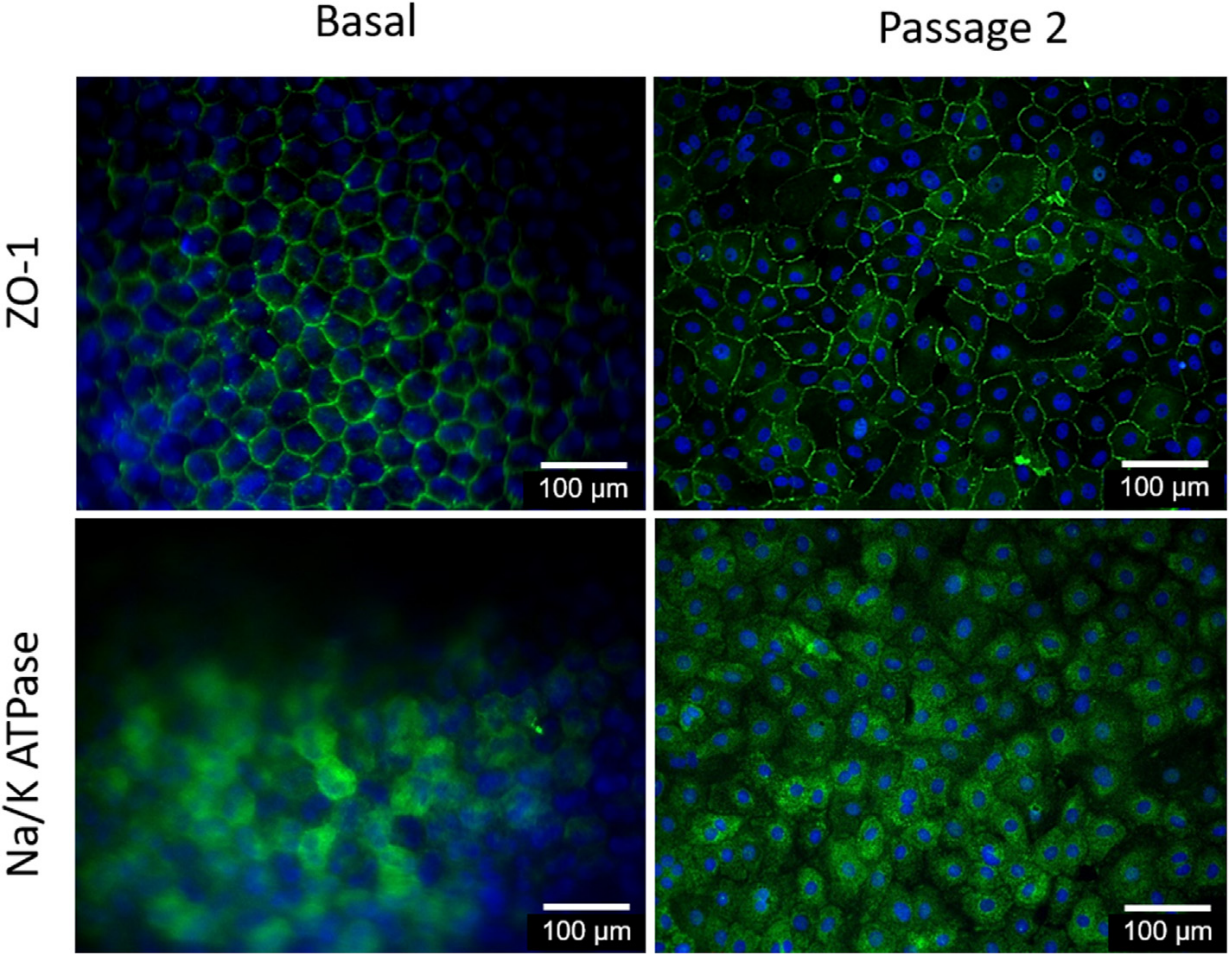
Merged images of immunocytochemistry of specific molecular markers of basal and cultured rabbit corneal endothelium cells with SQQQ combination. Basal corneal endothelium epifluorescence images of a whole cornea exhibit Zonula Occludens tight junctions (ZO-1) and Sodium–Potassium ATPase (Na+/K + -ATPase) expression. Also, cultured corneal endothelium cells exhibit specific molecular markers. Green secondary antibody: ZO-1 and Na+/K + -ATPase; Blue: Nuclei stained with DAPI.

Whole cornea immunofluorescence showed a basal state control to compare specific marker expression and location, as well as cell morphology (Figure 6). CEC cultured with SQQQ sequence expressed ZO-1 and Na^+^/K^+^-ATPase. Also, ZO-1 in basal CEC and those in passage 2 showed the expected hexagonal shape and expressed the location of the protein on the apical side [28]. Nevertheless, Na^+^/K^+^-ATPase basolateral location could not be assessed completely due to the swelling of stroma in whole cornea basal samples. Mitotic figures were observed at passage two; the implications will be discussed further on.

## 4 Discussion

### 4.1 Cell yield and morphology

All sequences showed a fold decrease with each passage as shown in Table 2. Nevertheless, the SQQQ sequence (Table 2; 4 and 6) maintained the fold number at passages 1 and 2. Also, our results on all resting media combinations QQQQ agree with previous reports of low proliferation and loss of circularity within passages [20]. Moreover, days to confluence increase with each passage, regardless of the media combination sequence as shown in Figure 2. This means media composition beyond the first passage is irrelevant.

Several authors have evaluated the effect of supplemented culture media to harvest CEC maintaining the hexagonal morphology and molecular characteristics [20, 27, 29, 30, 31]. Our results in rabbit cells in a two-phase culture system agree with the described in both, rabbit and human CEC [18, 20, 32] even though, our media lacks pituitary extract and ROCK inhibitors. The removal of pituitary extract obeys to the attempt of eliminating ingredients with a complex composition of animal origin; this way, the culture approach would be nearest to future human clinical applications. Furthermore, while media with pituitary extract is reported to promote proliferative capacity [15] it does not maintain the hexagonal morphology beyond the third passage [1, 13]. Also, in CEC culture, ROCK inhibitors induce fibroblast-like morphological changes [33, 34] and affect viability at 10, 30, and 100 μM [30]. Therefore, discarding their use and reaching the third passage, while conserving morphology represents an advantage for our research.

Moreover, this study has allowed us to understand that the modulation of cellular proliferation signaling produced by a two-phase culture system is only a prevention method for mesenchymal transition [24].

To evaluate morphology, shape descriptors circularity [18] and aspect ratio (length-to-width ratio) [30, 31] are commonly addressed indexes, when provided [14, 33]. Our study provides Circularity, Roundness, and Aspect Ratio tending to 1 at the end of passage 1 and 3. Our comparison between passages, and through time, allows us to observe the process of hexagonal morphology acquisition and establishment when confluence is reached. Also, we compared obtained circularity values with previously reported results [24]: 0.77 ± 0.063 SD for primary isolated rabbit CEC, 0.6 ± 0.18 for Quiescent, and 0.41 ± 0.19 for Stimulatory media. Based on those results, Stimulatory media produced the lowest circularity values, and with only two passages, it was detrimental to molecular expression; therefore, this combination was discarded. The average circularity value we now obtained, at the end of passage 3, with the SQQQ sequence is 0.78 ± 0.045 SD. Thus, our present SQQQ two-phase culture system improves previously obtained circularity, and proliferation is favored too, reaching a balance between morphology and cell yield [35].

In regards to the area and perimeter drop upon confluence, further are required to determine if a stabilization or compaction process occurs as observed in human CEC [4, 36, 37].

### 4.2 Total protein, pro-collagen I, Na^+^/K^+^-ATPase concentration and immunofluorescence

Total protein, pro-collagen I, and Na^+^/K^+^-ATPase concentrations were higher in CEC. A heterogeneous amount of CEC per group was evaluated due to their challenging isolation and culture. Nevertheless, in all protein determinations concentration resulted to be higher in CEC. Also, normalization to single-cell production was carried out for total protein and pro-collagen I to validate the comparison. CEC showed 3.2-fold higher protein concentration than control cells. Collagen I is one of the most abundant proteins produced by the corneal endothelium [38] whose arrangement is involved in corneal clarity. Procollagen I concentration was six times higher in CEC than in the control cells compared with a previous study that reports CEC higher ability of proline hydroxylation than most of the fibroblastic cell lines [39]. The Na^+^/K^+^-ATPase colorimetric analysis for CEC is a low cost and feasible methodology that results in useful to screen testing. The results support those of the immunocytochemistry analysis performed herein. Moreover, the functionality of the Na^+^/K^+^-ATPase is related to CEC hexagonal morphology procured with the SQQQ media combination and corroborated with the morphological analyses we provide [28].

Our previously reported results show Quiescent media sequences with immunofluorescence images alike those obtained with SQQQ. On the contrary, Stimulatory sequences show a less fluorescent signal for Na^+^/K^+^-ATPase and misplacement of the ZO-1 signal towards the cytoplasmic region of CEC [24]. Also, our findings on ZO-1 resemble those of Bartakova *et al.* [40], obtained using pituitary extract in the media. Nevertheless, Bartakova's Na^+^/K^+^-ATPase images appear completely different from ours and resemble more to those seen when we used Stimulatory sequences. However, our basal image (Figure 5) provides control for the desired protein expression.

Additionally, we found our results on ZO-1 and Na^+^/K^+^-ATPase immunofluorescence bear a resemblance to those of Peh *et al* [20] who also used a two-phase-media CEC culture method based on Stimulatory and Quiescent media alternation. Peh's M4 Stimulatory media lacks pituitary extract but, includes insulin, transferrin, and selenium. Also, the M5 Quiescent media includes human endothelial serum-free medium. Peh replaces the Stimulatory media with the Quiescent media when CEC reach 80–90% confluence and waits at least 7 days before passage. However, there are differences in media composition and culture process with our method, our results point out an improvement in the use of the present proposed model.

## 5 Conclusions

With the alternations of sequences of S media and Q media, we gathered evidence in favor of the use of dual culture media systems to improve CEC yield maintaining the hexagonal morphology. We discarded the use of pituitary extract and ROCK inhibitors as essential for CEC proliferation. Also, the SQQQ sequence demonstrated to be effective for CEC proliferation lowering the cost implied in SQSQ sequences with similar results. Finally, the findings on total protein, procollagen I, and Na^+^/K^+^-ATPase concentrations corroborate the immunocytochemistry evidence on the cellular identity of a highly organized and active metabolic monolayer tissue.

Sketching the limits of cultured CEC expansion is an unavoidable safety issue to be addressed to advance onto an *in-vivo* model. Our study allows guaranteeing cellular and tissular quality, as well as functionality for transplantation so further efforts might focus on improving surgery practices [41] and suggesting further monitoring techniques.

## Declarations

### Author contribution statement

M. Montalvo-Parra: Conceived and designed the experiments; Performed the experiments; Analyzed and interpreted the data; Wrote the paper.

I. Vidal-Paredes: Conceived and designed the experiments; Analyzed and interpreted the data; Wrote the paper.

C. Calzada-Rodríguez, I. Cárdenas-Rodríguez and G. Torres-Guerrero: Performed the experiments; Analyzed and interpreted the data.

D. Gómez-Elizondo and M. López-Martínez: Performed the experiments; Analyzed and interpreted the data.

J. Zavala: Analyzed and interpreted the data; Contributed reagents, materials, analysis tools or data; Wrote the paper.

J. Valdez-García: Analyzed and interpreted the data; Contributed reagents, materials, analysis tools or data.

### Funding statement

This work was supported by 10.13039/501100003141Consejo Nacional de Ciencia y Tecnología (CONACyT PN 6558) and 10.13039/501100004961Instituto Tecnologico y de Estudios Superiores de Monterrey.

### Competing interest statement

The authors declare no conflict of interest.

### Additional information

No additional information is available for this paper.
